# In Vivo Long‐Term Biodistribution, Excretion, and Toxicology of PEGylated Transition‐Metal Dichalcogenides MS_2_ (M = Mo, W, Ti) Nanosheets

**DOI:** 10.1002/advs.201600160

**Published:** 2016-05-27

**Authors:** Jiali Hao, Guosheng Song, Teng Liu, Xuan Yi, Kai Yang, Liang Cheng, Zhuang Liu

**Affiliations:** ^1^ Institute of Functional Nano & Soft Materials (FUNSOM)Collaborative Innovation Center of Suzhou Nano Science and TechnologySoochow UniversitySuzhouJiangsu215123China; ^2^ School of Radiation Medicine and Protection & Radiological and Interdisciplinary Sciences (RAD‐X)Jiangsu Provincial Key Laboratory of Radiation Medicine and ProtectionSoochow UniversitySuzhouJiangsu215123China

**Keywords:** biodistribution, degradation, excretion, TMDCs, toxicology

## Abstract

With unique 2D structures and intriguing physicochemical properties, various types of transition metal dichalcogenides (TMDCs) have attracted much attention in many fields including nanomedicine. Hence, it is of great importance to carefully study the in vivo biodistribution, excretion, and toxicology profiles of different TMDCs, and hopefully to identify the most promising type of TMDCs with low toxicity and fast excretion for further biomedical applications. Herein, the in vivo behaviors of three representative TMDCs including molybdenum dichalcogenides (MoS_2_), tungsten dichalcogenides (WS_2_), and titanium dichalcogenides (TiS_2_) nanosheets are systematically investigated. Without showing significant in vitro cytotoxicity, all the three types of polyethylene glycol (PEG) functionalized TMDCs show dominate accumulation in reticuloendothelial systems (RES) such as liver and spleen after intravenous injection. In marked contrast to WS_2_‐PEG and TiS_2_‐PEG, which show high levels in the organs for months, MoS_2_‐PEG can be degraded and then excreted almost completely within one month. Further degradation experiments indicate that the distinctive in vivo excretion behaviors of TDMCs can be attributed to their different chemical properties. This work suggests that MoS_2_, among various TMDCs, may be particularly interesting for further biomedical applications owning to its low toxicity, capability of biodegradation, and rapid excretion.

## 1 Introduction

In the past few years, 2D nanomaterials have received tremendous attention and been applied in various fields due to their fantastic physical and chemical properties.^1^, ^2^, ^3^, ^4^, ^5^, ^6^, ^7^, ^8^ As an analogue of 2D graphene, transition metal dichalcogenides (TMDCs), generally described as the formula MX_2_, in which M is the transition metal from groups 4–10 of the periodic table and X is a chalcogen (S, Se, or Te), have attracted great interests due to their rich electronic, optical, mechanical, and chemical properties.^4^, ^9^, ^10^, ^11^, ^12^, ^13^ In particular, different combinations of transition metals and chalcogens, as well as their various arrangements in the 2D crystals, would lead to a substantial range of properties, making TMDC materials interesting for applications in biological systems. For example, many biosensing platforms have been fabricated based on TMDCs for detection of biological molecules.^14^, ^15^ The high near infrared (NIR) absorbance of selected 2D TMDCs, such as molybdenum dichalcogenides (MoS_2_), tungsten dichalcogenides (WS_2_), and titanium dichalcogenides (TiS_2_), have made them ideal agents for photothermal therapy.^16^, ^17^, ^18^, ^19^ The extraordinary surface‐area‐to‐mass ratio of single layered 2D TMDCs affords them high drug loading capacity useful for drug delivery and combination cancer therapy.^20^, ^21^ TMDCs and their doped or composite nanostructures have also shown great promises as novel contrast agents for multimodal biomedical imaging.^22^, ^23^, ^24^, ^25^ Additionally, TMDCs with interesting mechanical properties have been explored in the area of tissue engineering.^26^ Considering the great potential of TMDCs in biomedical applications, it is therefore critically important to systematically evaluate their long‐term in vivo behaviors.

In the past few years, there have been a number of reports studying the toxicity of TMDCs nanomaterials in vitro and in vivo.^27^, ^28^, ^29^, ^30^, ^31^ At the in vitro level, it was found that the cytotoxicity of MoS_2_, WS_2_, and WSe_2_ nanosheets appeared to be low to different cell lines.^15^, ^18^, ^32^, ^33^ On the other hand, in our previous reports about the use of polyethylene glycol (PEG) functionalized TMDCs for in vivo imaging and therapy applications, we have uncovered that PEGylated MoS_2_ and WS_2_ showed no appreciable acute toxicity to the treated mice at our tested dose.^16^, ^20^, ^23^, ^34^ Similar findings have also been reported by several other groups regarding the in vivo toxicity of TMDCs with biocompatible surface coatings.^21^, ^30^, ^35^ However, the detailed in vivo long‐term degradation and excretion behaviors of various TMDCs remain to be investigated to our best knowledge. In addition, it would be very interesting and important to find out which type of TMDCs has the fastest excretion and least long‐term retention, and thus could be the most promising one for further in vivo biomedical applications.

In this work, we have synthesized three representative TMDCs (MoS_2_, WS_2_, and TiS_2_) by a high‐temperature solution‐phase method and then broke them into small nanosheets under ultra‐sonication. After functionalization with PEG, these three types of PEGylated TMDCs nanosheets showed great physiological stability and quite low in vitro cytotoxicity. Healthy mice were intravenously (*i.v*.) injected with MS_2_‐PEG (M = Mo, W, Ti) and then sacrificed at different time points with major organs collected. All the three types of MS_2_‐PEG nanosheets showed dominant accumulation in reticuloendothelial systems (RES) such as liver and spleen. Interestingly, while high levels of W and Ti were detected in RES organs of mice post injection of WS_2_‐PEG and TiS_2_‐PEG, respectively, even after 30 d, we found that MoS_2_‐PEG could be excreted from the body almost completely within 30 d by urine and feces, likely owing to the oxidization of Mo^IV^S_2_ into water‐soluble Mo^VI^‐oxide species (e.g., MoO_4_^2−^). The blood analysis and histological examination of those mice showed no obvious in vivo toxicity of all three types of PEGylated TMDCs at our tested dose, even for WS_2_‐PEG and TiS_2_‐PEG with long‐term retention in mouse RES organs. Our work suggests that MoS_2_ could be a promising 2D nanomaterial for biomedical applications due to its biodegradability and relatively fast excretion.

## 2 Results and Discussion

### 2.1 Synthesis and Surface Modification of MoS_2_, WS_2_, and TiS_2_ Nanosheets

**Figure 1:**
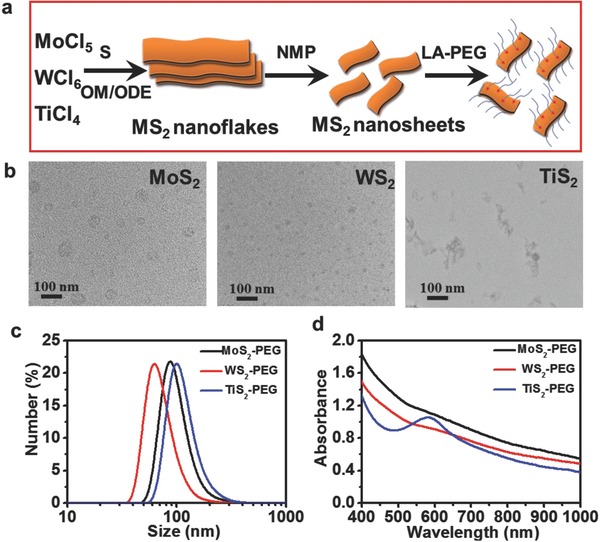
Synthesis and characterization of PEGylated MS_2_ (M = Mo, W, Ti) nanosheets. a) A scheme of MS_2_ TMDC nanosheets synthesis process. b) TEM images of PEGylated MoS_2_, WS_2_, and TiS_2_ nanosheeets. c) DLS size distribution of PEGylated MoS_2_, WS_2_, and TiS_2_ nanosheets in water. d) UV–vis‐NIR absorbance spectra of PEGylated MosS_2_, WS_2_, and TiS_2_ nanosheets with the concentration of 0.02 mg mL^−1^.

TMDCs (MoS_2_, WS_2_, and TiS_2_) nanoflakes were synthesized by a high‐temperature solution‐phase method, TEM images (Figure S1, Supporting Information) have showed the morphologies of MoS_2_, WS_2_, and TiS_2_ nanoflakes. Then the synthesized MoS_2_, WS_2_, and TiS_2_ were dissolved in 1‐methyl‐2‐pyrrolidinone (NMP) and broken into small and single‐layer nanosheets under ultrasonication^36^ (**Figure** **1**a). In brief, the metal precursors (MoCl_5_, WCl_6_, and TiCl_4_) were first reacted with oleylamine (OM) to obtain M‐OM complexes (M = Mo, W, and Ti). Upon injection of the sulfur solution by dissolving sulfur powder in OM, the solution color immediately turned into black or deep brown, suggesting the rapid formation of TMDC nanoflakes. Transmission electron microscope (TEM) image (Figure S1, Supporting Information) indicated that the synthesized TMDCs exhibited flake‐like structures. Under ultrasonication in NMP solution, all TMDCs nanoflakes were broken into smaller nanosheets, which became soluble in water. The phase analysis of the as‐prepared nanosheets was also determined by power X‐ray diffraction (XRD) (Figure S2, Supporting Information). All peaks in these spectra matched well with the reported results.^16^, ^17^, ^20^

After ultrasonication in NMP, the as‐synthesized MoS_2_/WS_2_/TiS_2_ nanosheets became water‐soluble, we have tested the dynamic light scattering (DLS) size distribution of these three nanosheets in water (Figure S3, Supporting Information). Since the as‐made MoS_2_/WS_2_/TiS_2_ presented good water‐solubility but bad stability in physiological solutions in the presence of salts. We then chose lipoic acid conjugated 5 kDa PEG (LA‐PEG_5k_) to modify MoS_2_/WS_2_/TiS_2_ nanosheets through M‐S (M = Mo, W, Ti) bond. Thermogravimetric analysis showed the weight percentages of MoS_2_, WS_2_, and TiS_2_ in the PEGylated samples were determined to be 25.99%, 59.72%, and 46.92% (Figure S4, Supporting Information), respectively. After PEGylation, the hydrodynamic diameters of MoS_2_‐PEG, WS_2_‐PEG, and TiS_2_‐PEG were determined by DLS to be ≈91, ≈72, and ≈102 nm (Figure 1c), respectively. We also have collected the statistics of TEM measured sizes about PEGylated MoS_2_, WS_2_, and TiS_2_ (Figure S5, Supporting Information). Through the TEM images we could find that the diameters of PEGylated MoS_2_/WS_2_/TiS_2_ were smaller compared with MoS_2_/WS_2_/TiS_2_ nanosheets, which could be attributed to the functionalization process. MS_2_ were synthesized by a high‐temperature solution‐phase method and then broken in NMP under ultrasonication to obtain small nanosheets. After that, the small nanosheets were functionalized by LA‐PEG under ultrasonication in the water phase. The modification process would further broke the nanosheets, which lead smaller size compared with MS_2_ under TEM.

All PEGylated TMDC nanosheets were rather stable in various physiological solutions including phosphate buffered saline (PBS), RPMI‐1640 medium, and fetal bovine serum (Figure S6, Supporting Information). UV–vis‐NIR spectra of PEGylated TMDC nanosheets all showed strong wide‐band NIR absorbance (Figure 1d), with the weight extinction coefficients of MoS_2_‐PEG, WS_2_‐PEG, and TiS_2_‐PEG measured to be 26.36, 23.42, and 22.04 Lg^−1^ cm^−1^, respectively, at 808 nm.

### 2.2 In Vitro Cytotoxicity Study for PEGylated TMDCs

**Figure 2:**
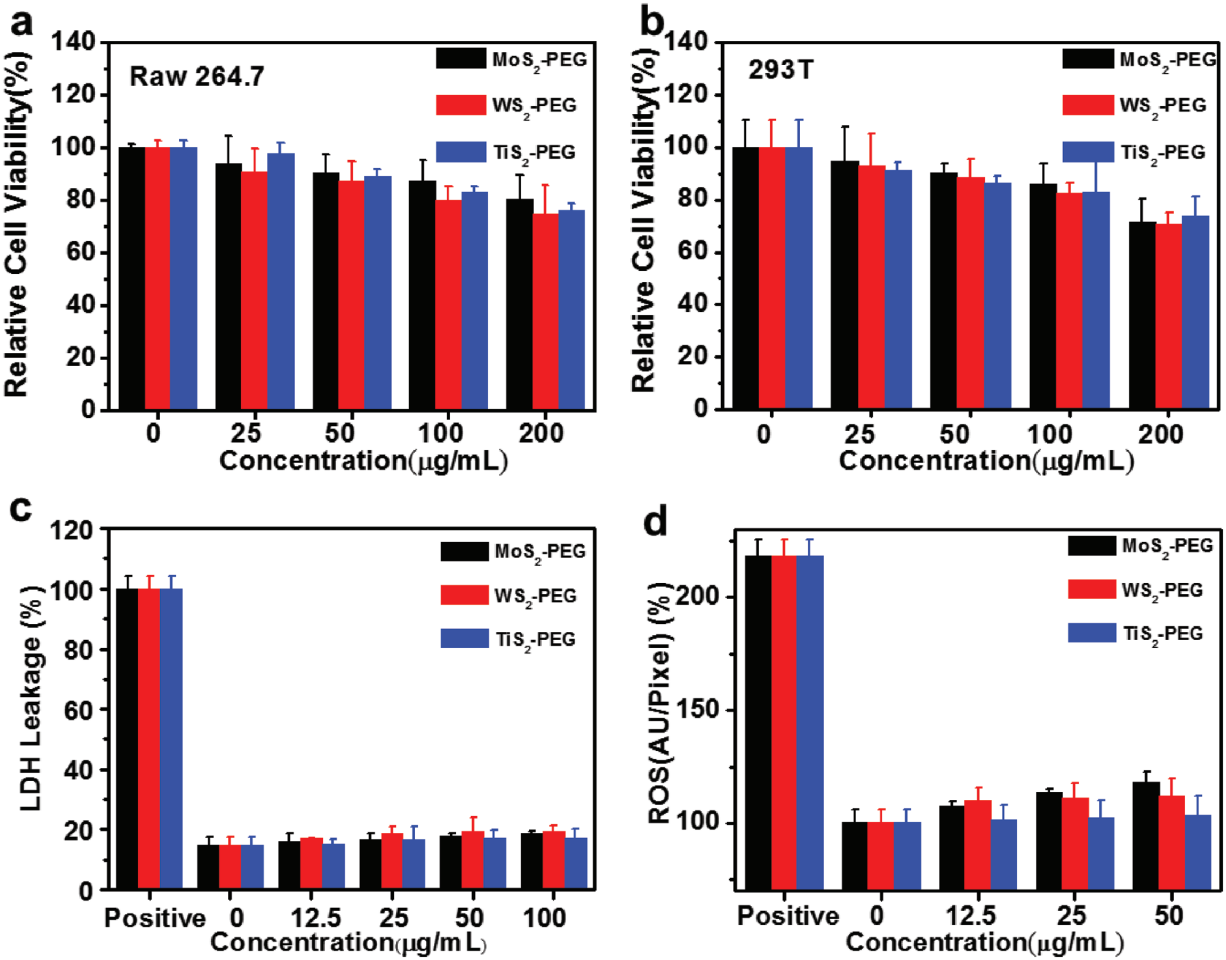
Cytotoxicity of PEGylated MS_2_ (M = Mo, W, Ti). a,b) Relative cell viability of Raw 264.7 cell line (a) and 293T cell line (b) after incubation with PEGylated MoS_2_, WS_2_, and TiS_2_ at different MS_2_ weight concentrations for 24 h. c,d) Relative LDH release level (c) and relative ROS values (d) of 4T1 cell line after incubation with PEGylated MoS_2_, WS_2_, and TiS_2_ nanosheets at different concentrations for 24 h, respectively.

We then carried out a number of different assays to study the in vitro cytotoxicity of various MS_2_‐PEG (M = Mo, W, and Ti) samples. First, the standard 3‐(4,5‐dimethylthiazol‐2‐yl)‐2, 5‐diphenyltetrazolium bromide (MTT) assay was performed on mouse macrophage Raw 264.7, human renal epithelial cell 293T and mouse breast cancer 4T1 cell lines (Figure S7, Supporting Information). After incubation with different concentrations of PEGylated MoS_2_, WS_2_, and TiS_2_ for 24 h, we did not find any serious cytotoxicity for these three kinds of MS_2_‐PEG nanosheets to both two cell lines even at high concentrations (e.g., 200 μg mL^−1^, in terms of MS_2_ weight concentrations) (**Figure** **2**a,b). Next, the lactate dehydrogenase (LDH) leakage assay was performed to test the integrity of cell membrane after cells were exposed to PEGylated TMDCs for 24 h (Figure 2c).^37^ It was found that the all these three MS_2_‐PEG (M = Mo, W, and Ti) showed little damage to cells even at high concentrations. When cells are under stimulation by foreign damage signals, the oxidative stress inside cells may be increased to activate the cellular defense system.^38^ Herein, we used a dihydroethidine (DHE) probe to detect the intracellular reactive oxygen species (ROS, e.g., peroxide and superoxide) in cells after incubation with MS_2_‐PEG (Figure 2d). Compared with the control group, the experimental groups also showed no noticeable increase of intracellular ROS generation after treatment by MS_2_‐PEG. All these data put together indicated that our PEGylated MoS_2_, WS_2_, and TiS_2_ nanosheets exhibited no appreciable in vitro cytotoxicity within our tested dose range.

### 2.3 In Vivo Biodistribution and Clearance Behaviors of PEGylated TMDCs

**Figure 3:**
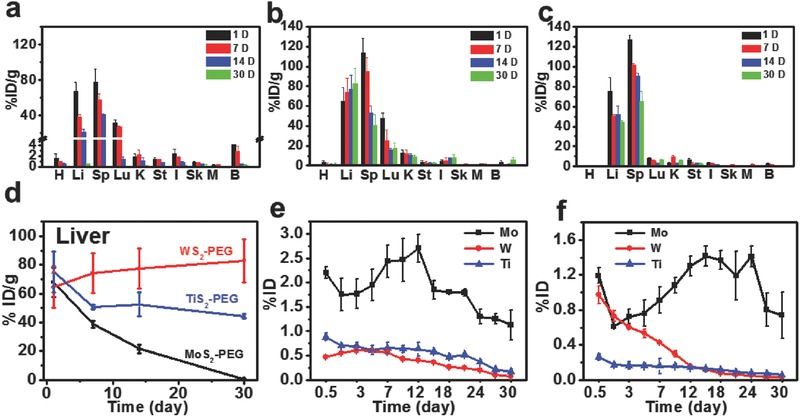
In vivo biodistribution and excretion of PEGylated MS_2_ (M = Mo, W, Ti). a–c) Biodistribution of MoS_2_‐PEG (a), WS_2_‐PEG (b), and TiS_2_‐PEG (c) in different organs after *i.v* injection (H: heart; L: liver; Sp: spleen; Lu: lung; K: kidney; St: stomach; I: intestine: Sk: skin; M: muscle; and B: bone). d) The clearance effect of liver after *i.v* injection of PEGylated MoS_2_, WS_2_, and TiS_2_ at different time points. e,f) Urinary excretion (e) and fecal excretion (f) of PEGylated MoS_2_, WS_2_, and TiS_2_ at different time points. Error bars in the above data were based on standard deviations of four mice per group.

The biodistribution and clearance behaviors of nanomaterials are highly important for their potential use in biomedicine. However, to our best knowledge, systematic studies to investigate the long‐term in vivo behaviors of various TMDCs in parallel are still missing to date. In this work, healthy Balb/c mice were *i.v*. injected with PEGylated MoS_2_, WS_2_, and TiS_2_ (10 mg kg^−1^, in terms of TMDC weight concentrations). Their major organs including heart (H), liver (L), spleen (Sp), lung (Lu), kidney (K), stomach (St), intestine (I), skin (Sk), muscle (M), and bone (B) were collected at different time points post injection and then split into two halves, one for biodistribution study and the other for histology examination. For biodistribution measurement, the organs were weighted and digested by aqua regia. The contents of Mo, W, and Ti were measured by inductively coupled plasma‐atomic emission spectrometry (ICP‐AES) (**Figure** **3**a–c). It was found that all three types of PEGylated TMDCs accumulated mostly in RES organs such as liver and spleen at 1 d post injection (*p.i*), as the results of phagocytose by the Kupffer cells and spleen macrophages in these two organs. At later time points, however, we found that the metabolic rate of MoS_2_‐PEG was much faster than that of WS_2_‐PEG and TiS_2_‐PEG. After 30 d, the detected Mo levels decreased sharply to 0.54 ± 0.069 % ID g^−1^ in the liver (from 67.74 ±10.26% ID g^−1^ at day 1) and 0.017 ± 0.015% ID g^−1^ in the spleen (from 77.70 ±14.91% ID g^−1^ at day 1), with most of Mo excreted from the body (Figure 3a). Different from MoS_2_‐PEG, the other two types of TMDC nanosheets showed much slower excretion, with rather high levels of W and Ti levels detected in the liver and spleen after 30 d (Figure 3b,c). Figure 3d showed the Mo, W, and Ti levels in the liver at various time points. It was found that MoS_2_‐PEG showed remarkably faster excretion from this organ compared to the other two types of PEGylated TMDCs. Unexpectedly, the W content in the liver increased slightly over time in mice injected with WS_2_‐PEG, likely owing to the gradual translocation of WS_2_ into liver from spleen, in which the W content significantly dropped, as well as the rather slow excretion of WS_2_ from the liver.

In order to further investigate the clearance pathway, Balb/c mice after *i.v* injection with PEGylated TMDCs were housed in metabolic cages to collect their urine and feces, which were then digested by aqua regia to measure metal ion contents using ICP‐AES. Interestingly, high concentrations of Mo were detected in both urine and feces from MoS_2_‐PEG injected mice, while quite low levels of W and Ti were found in the urine and feces of mice injected with the other two types of PEGylated TMDCs (Figure 3e,f). Those results further confirmed that MoS_2_‐PEG, unlike the other two types of TMDCs, could be excreted effectively via both renal and fecal pathways.

**Figure 4:**
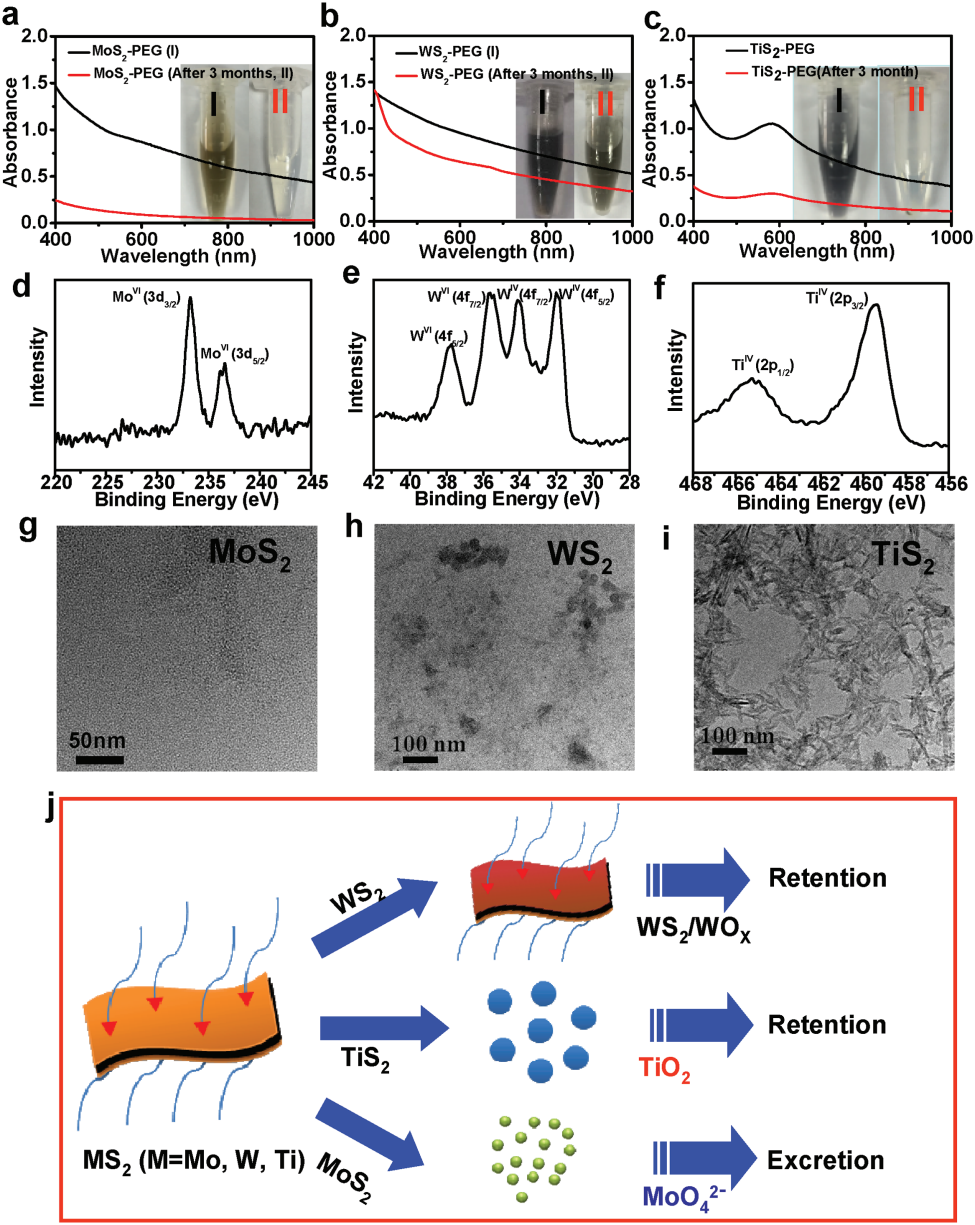
Mechanism of the clearance of the MS_2_‐PEG nanosheets (M = Mo, W, and Ti). a–c) UV–vis‐NIR spectra of MS_2_‐PEG (**I**) before and after three months (**II**) standing in PBS at the concentration of 0.02 mg mL^−1^, (a) MoS_2_‐PEG, (b) WS_2_‐PEG, and (c) TiS_2_‐PEG. Inset: Photos of the MS_2_‐PEG (M = Mo, W, and Ti) samples before (**I**) and after three months (**II**) standing in PBS solution. d–f) XPS spectra of the Mo^VI+^ (d), W^VI+^ and W^IV+^ after oxidation (e), and Ti^IV^ of TiO_2_ nanoparticles from TiS_2_‐PEG after three months standing in PBS (f). g–i) TEM images of the PEGylated MoS_2_ (g), WS_2_ (h), and TiS_2_ (i) nanosheets after three months standing. j) A scheme showing the different pathways of the clearance of MS_2_‐PEG nanosheets (M = Mo, W, and Ti).

A serial of experiments were next carried out to find out the mechanism of different metabolism behaviors for these three TMDCs. Since the phosphate buffer saline (PBS) is the most frequently used physiological buffer, we have chosen PBS buffer for the in vitro degradation experiments. PEGylated MoS_2_, WS_2_, and TiS_2_ were dissolved in PBS at room temperature for three months. Compared to WS_2_‐PEG which only showed slightly decreased UV–vis‐NIR absorbance after storage for three months, the optical absorbance of both MoS_2_‐PEG and TiS_2_‐PEG samples decreased remarkably (**Figure** **4**a–c). Notably, while a transparent colorless solution was left for MoS_2_‐PEG after incubation for three months, white precipitate was observed in the TiS_2_‐PEG sample (inset of Figure 4a–c). We then used X‐ray photoelectron spectroscopy (XPS) to analyze different TMDC samples dissolved in PBS for three month. It could be found that most of Mo element in MoS_2_ nanosheets was oxidized to the high valence state (Mo^VI^) (Figure 4d), suggesting the oxidization of Mo^IV^S_2_ with dark brown color into colorless water‐soluble Mo^VI^‐oxide species (e.g. MoO_4_^2−^). Different from MoS_2_, only a part of high valence state of W (W^VI^) was observed for the WS_2_‐PEG sample after three month storage in PBS (Figure 4e), suggesting the formation of W^IV^S_2_/W^VI^O_3_ compounds after such incomplete oxidation. Such a difference between MoS_2_‐PEG and WS_2_‐PEG could probably be due to the better chemical stability of W^IV^ and stronger chemical band of W‐S in WS_2_ compared to the counterparts in MoS_2._ 39 As for the TiS_2_‐PEG sample, the insoluble white precipitate seen in the bottom of Ependor tube was found to be TiO_2_ based on both XPS (Figure 4f**)** and XRD data **(**Figure S8, Supporting Information). TEM images of these three samples also showed that while no large nanoparticles were found in the degradation product of MoS_2_‐PEG, lots of aggregates were formed for another two samples (WS_2_‐PEG and TiS_2_‐PEG, Figure 4g–i).

Based on the above observations, we conclude that the different in vivo excretion behaviors of three types of TMDCs should be due to their distinctive chemical properties (Figure 4j). WS_2_ shows relatively high stability in the physiological environment, and can hardly be degraded. Therefore WS_2_‐PEG after *i.v*. injection would retain in RES organs for a long time without rapid excretion. TiS_2_ is not stable and could be gradually oxidized into water‐insoluble TiO_2_ aggregates, which however also could not be easily excreted from the mouse body. In marked contrast, MoS_2_ within the physiological environment could be oxidized and transformed into water‐soluble Mo^VI^‐oxide species (e.g., MoO_4_^2−^), which are then readily excreted from the mouse body via both renal and fecal pathways.

### 2.4 In Vivo Toxicology Study of PEGylated MoS_2_, WS_2_, and TiS_2_

**Figure 5:**
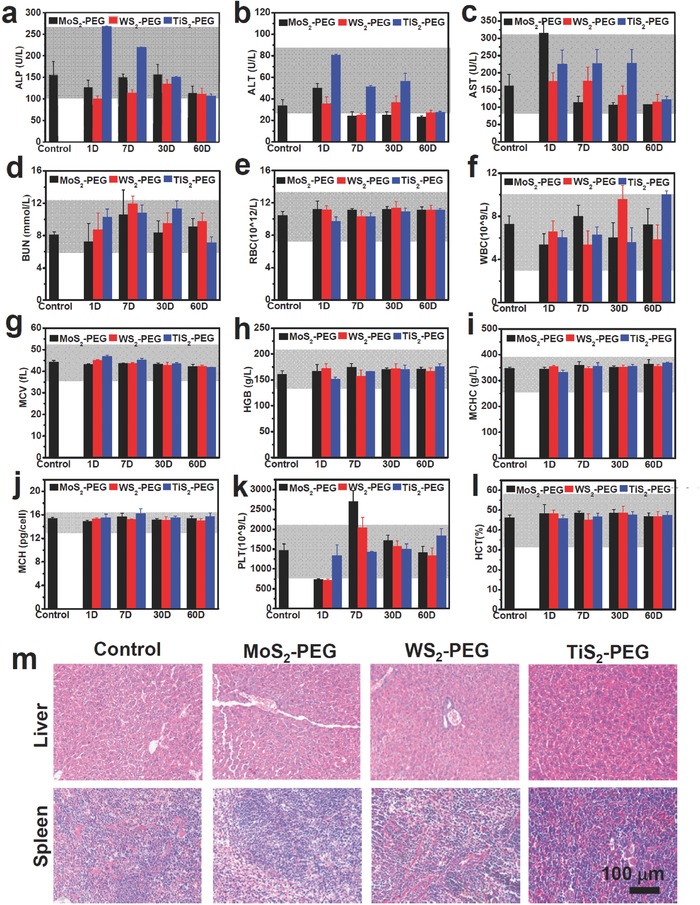
Blood biochemistry and hematology data of Balb/c mice treated with MS_2_‐PEG nanosheets (M = Mo, W, and Ti). The data were collected at different time point after *i.v* injection, error bars were based on standard deviations of four mice per group. a) Alkaline phosphatase (ALP), b) alanine aminotransferase (ALT), c) aspartate aminotransferase (AST), and d) urea nitrogen (BUN) levels in the blood at different time point. e) Red blood cells (RBC), f) white blood cells (WBC), g) mean corpuscular volume (MCV), h) hemoglobin (HGB), i) mean corpuscular hemoglobin concentration (MCHC), j) mean corpuscular hemoglobin (MCH), k) platelet (PLT), and l) hematocrit (HCT) levels in the blood at different time point. Gray areas in the figures show the normal reference ranges of hematology data of male Balb/c mice. m**)** H&E staining images of liver and spleen from mice treated with MS_2_‐PEG nanosheets (M = Mo, W, and Ti). The organs were collected from the mice sacrificed at 30 d post injection. The control group was obtained from the untreated mice.

At last, we carefully looked into whether PEGylated TMDCs would exert any toxic effect to mice, by both hematology assay and histology examination. For the same female Balb/c mice used for biodistribution studies, their blood was collected at 1, 7, 30, 60 d post *i.v* injection of MS_2_‐PEG (M = Mo, W, and Ti). Various serum biochemical parameters including alkaline phosphatase (ALP), alanine aminotransferase (ALT), aspartate aminotransferase (AST), and urea nitrogen (BUN) were measured (**Figure** **5**a–d). After 1 d treatment with MoS_2_‐PEG, all the serum biochemical parameters were close to control group except AST, which increased at 1 d p.i. but dropped 7 d later. The biochemical parameters for the WS_2_‐PEG treated group showed no obvious difference compared with untreated mice. As for the TiS_2_‐PEG treatment group, the ALT and ALP activities in plasma were increased compared to the control group. However, all these parameters decreased into the control levels later, demonstrating no irreversible injury to the liver of mice induced by TiS_2_‐PEG. Notably, all those variations were still within the reference ranges for healthy Balb/c mice.

For the blood routine examination, red blood cells (RBC), white blood cells (WBC), hemoglobin (HGB), mean corpuscular volume (MCV), mean corpuscular hemoglobin concentration (MCHC), hematocrit (HCT), mean corpuscular hemoglobin (MCH), and platelet (PLT) counts were measured (Figure 5e–l). All the parameters tested in the treated groups during the monitoring period were within the reference normal ranges,^40^ except some variations in platelet counts. The decrease in the platelet count 1 d after treatment may be due to the absorption of TMDCs nanosheets on blood cells, similar to the behavior of some other nanomaterials previously reported.^41^, ^42^ Later on, the platelet count increased rapidly to the normal range.

While the first halves of organs were used for biodistribution studies, the other halves of major organs from mice injected with different PEGylated TMDCs were sliced for hematoxylin and eosin (H&E) staining and histological examination. No obvious sign of abnormality, such as inflammation, was noticed in all examined major organs, including liver and spleen with domination accumulation of those nanomaterials (Figure 5m**)**. Our results suggest that all of the three PEGylated TMDCs nanosheets have no significant toxicity to mice within 30 d. In particularly, considering the almost complete clearance of MoS_2_‐PEG within 30 d, it is reasonably to predict that MoS_2_‐PEG would not cause further long‐term toxicity to the treated animals in a reasonable dose range.

## 3 Conclusions

In summary, we have systemically studied the in vivo biodistribution, excretion, and toxicology profiles of three respective types of TMDCs with surface PEGylation. The biodistribution results indicated abundant accumulation of those nanosheets in RES organs after *i.v* injection. Notably, after 30 d, MoS_2_‐PEG could be excreted almost completely, while large amounts of injected W or Ti were still retained in the mouse RES organs for those injected with WS_2_‐PEG or TiS_2_‐PEG, respectively. Further degradation experiments uncovered that MoS_2_ could be oxidized into water‐soluble Mo^VI^‐oxide species (e.g., MoO_4_^2−^) to allow its rapid clearance from the body. In contrast, WS_2_ with much higher chemical stability was more difficult to be oxidized and thus showed much longer in vivo retention, while TiS_2_ could be oxidized to into water‐insoluble TiO_2_ aggregates that were also hard to be excreted. Moreover, further histological and blood analysis showed no obvious long‐term toxicity of these three types of TMDC nanosheets at our tested dose. This work is the first time to carefully compare the in vivo biodistribution, degradation, and excretion behaviors of different TMDCs side‐by‐side. Our results suggest that MoS_2_, among various TMDC nanomaterials, may be particularly promising for further biomedical applications owning to its biodegradability and relatively rapid excretion.

## 4 Experiment Section

*Materials*: All the chemical reagents, unless specified, were purchased and used without further purification. Tungsten (VI) chloride (WCl_6_), molybdenum (V) chloride (MoCl_5_), titanium tetrachloride (TiCl_4_), oleylamine (OM), 1‐octadecene (ODE), and NMP were purchased from Sigma‐Aldrich. Sulfur powder (S) was obtained from Sinopharm Chemical Reagent Co., Ltd. (China). Lipoic acid was purchased from Sigma‐Aldrich and mPEG‐NH_2_ (MW = 5K) were obtained from Biomatrik Co., Ltd (Jiaxing, China). Deionized water used in our experiments was obtained by using a Milli‐Q water system.

*Synthesis of MoS_2_, WS_2_*, *and TiS_2_ Nanosheets Synthesis*: First, MoS_2_, WS_2_, and TiS_2_ nanoflakes were synthesized according the previous report with slight modification.^19^, ^36^ 1 mmol MoCl_5_/WCl_6_ TiCl_4_/was dissolved in 15 mL oleylamine (OM) and 10 mL 1‐octadecene (ODE) in a three‐necked flask under magnetic stirring, then heated to 150 °C with the protection of nitrogen atmosphere and kept for 20 min to remove the low‐boiling‐point impurities. Then the temperature was increased to 300 °C slowly. 2.5 mmol sulfur powder dissolved in 4 mL OM was injected to the reaction solution rapidly. The solution temperature was kept at 300 °C for another 30 min and then cooled down to the room temperature. The product was dissolved in cyclohexane and washed with ethyl alcohol/cyclohexane (V:V = 1:1) for three times. After that, as‐synthesized MoS_2_, WS_2_, and TiS_2_ nanoflakes were dissolved in NMP solution, and sonicated for 5 h using a water‐bath ultra‐sonicator. The nanosheets were precipitated by centrifugation with ethyl alcohol and then dissolved in DI water.

*Functionalization of MoS_2_, WS_2_, and TiS_2_ Nanosheets*: LA‐PEG was synthesized following a literature procedure.^43^ 20 mg LA‐PEG was added to 10 mL aqueous solution of MoS_2_, WS_2_, or TiS_2_ (1 mg mL^−1^) under magnetic stirring at room temperature overnight. Then the solution was centrifuged 4000 rpm for 20 min by Amicon filters (Millipore) (MWCO = 10 kDa) and washed with DI water to remove excess polymer, obtaining PEGylated MoS_2_, WS_2_, and TiS_2_.

*Characterization*: The TEM images were taken by a FEI Tecnai F20 TEM. XRD measurement was performed by a PANalytical X‐ray diffractometer at Cuka radiation (λ = 0.15406 nm). UV–vis‐NIR spectra of PEGylated MoS_2_, WS_2_, and TiS_2_ were acquired by a PerkinElmer Lambda 750 UV–vis‐NIR spectrophotometer. The sizes of PEGylated MoS_2_, WS_2_, and TiS_2_ were measured by dynamic light scattering (MALVERN ZEN3690). The absolute Mo/W/Ti contents as well as MS_2_ weight concentrations in different MS_2_‐PEG samples were measured by ICP‐AES (Vista Mpx 700‐ES).

*In Vitro Cytotoxicity Assay*: The mouse macrophage RAW 264.7 cells, murine breast cancer 4T1 cells, and human renal epithelial cell 293T were chosen to evaluate the cytotoxicity of PEGylated MoS_2_, WS_2_, and TiS_2_ nanosheets. Both cells were originally obtained from American Type Culture Collection (ATCC) and cultured at 37 °C within 5% CO_2_ in RPMI‐1640 cell medium supplemented with 10% fetal bovine serum (FBS). For the MTT assay, the cells were seeded in 96‐well plates and incubated with PEGylated MoS_2_, WS_2_, and TiS_2_ at different concentrations. 24 h later, the cells were washed with fresh medium and preceded to the standard MTT assay or LDH assay following vendors' protocols. For ROS detection, cells after treatment with PEGylated MoS_2_, WS_2_, and TiS_2_ for 24 h were incubated with dihydroethidine (DHE) probe for 40 min. Then cells were collected, washed with PBS twice, and preceded to flow cytometry analysis (FACSCalibur, BD).

*Biodistribution Study*: Female Balb/c mice (20 g ± 2 g) were bought from Nanjing Peng Sheng Biological Technology Co. Ltd and used under protocols approved by Soochow University Laboratory Animal Center. Balb/c mice were *i.v* injected with PEGylated MoS_2_, WS_2_, and TiS_2_ (200 μL per mouse, 1 mg mL^−1^, dosage = 10 mg kg^−1^, in terms of MS_2_ weight concentrations). Control group were *i.v* injected with 200 μl PBS. Four mice were used per group. The mice were sacrificed with major organs including heart, liver, spleen, lung, kidney, stomach, intestine, skin, muscle, bone collected at day 1, day 7, day 14, day 30, and day 60 *p.i*. Those organs were added with 10 mL aqua regia (HCl:HNO_3_:HClO_4_ = 3:1:1) and heated to 200 °C for 2 h. After being cooled down to room temperature, each sample was diluted to 10 mL by DI water and passed through a 0.22 μm filter to remove any undigested tissue. The amount of Mo, W, and Ti were measured by ICP‐AES (Vista Mpx 700‐ES).

*Excretion Study*: Female Balb/c mice were *i.v* injected with PEGylated MoS_2_, WS_2_, and TiS_2_ (dosage = 10 mg kg^−1^) with four mice per group. Each mouse was placed in metabolism cage. Their urine and feces were collected at regular time points. The metabolism cages were washed after each round of collection. The collected urine and feces were digested with aqua regia for ICP‐AES measurement of Mo, W, and Ti contents (Vista Mpx 700‐ES).

*Toxicology Study*: Female Balb/c mice were *i.v* injected with PEGylated MoS_2_, WS_2_, and TiS_2_ (dosage = 10 mg kg^−1^, in terms of MS_2_ weight concentrations) with four mice per group. At 1, 7, and 30 d p.i., blood from those mice were drawn through their orbital venous plexus. For the blood chemistry analysis, alkaline phosphatase (ALP), alanine aminotransferase (ALT), aspartate aminotransferase (AST), and urea nitrogen (BUN) were measured. For the complete blood panel test, red blood cells, white blood cells, hemoglobin, mean corpuscular volume, mean corpuscular hemoglobin concentration, mean corpuscular hemoglobin, hematocrit, and platelet count were measured. All the assays were tested in Shanghai Research Center for Biomodel Organism. For histology study, heart, liver, spleen, lung, kidney collected from those mice were fixed in 10% neutral buffered formalin, processed routinely into paraffin, sectioned into 8 μm thickness slices, and then stained with hematoxylin & eosin (H&E). The slices were observed with a digital microscope (Leica QWin).

## Supporting information

As a service to our authors and readers, this journal provides supporting information supplied by the authors. Such materials are peer reviewed and may be re‐organized for online delivery, but are not copy‐edited or typeset. Technical support issues arising from supporting information (other than missing files) should be addressed to the authors.

Supplementary
