# Supplementary material for: In Vivo Long‐Term Biodistribution, Excretion, and Toxicology of PEGylated Transition‐Metal Dichalcogenides MS2 (M = Mo, W, Ti) Nanosheets
Source: Adv Sci (Weinh). 2016 May 27;4(1):1600160. doi: 10.1002/advs.201600160 (PMC5238746; doi:10.1002/advs.201600160)
Supplement: Supplementary file 1 — Supplementary [file ADVS-4-0-s001.pdf]

Copyright WILEY-VCH Verlag GmbH & Co. KGaA, 69469 Weinheim, Germany, 2016.

ADVANCED SCIENCE Open Access

Supporting Information

for *Adv. Sci.,* DOI: 10.1002/advs.201600160

In Vivo Long-Term Biodistribution, Excretion, and Toxicology of PEGylated Transition-Metal Dichalcogenides MS_2 (M = Mo, W, Ti) Nanosheets

Jiali Hao, Guosheng Song, Teng Liu, Xuan Yi, Kai Yang, Liang Cheng, and Zhuang Liu**

Supporting Information

In Vivo Long-Term Biodistribution, Excretion and Toxicology of PEGylated Transition-Metal Dichalcogenides MS_2 (M=Mo, W, Ti) Nanosheets

Jiali Hao[1], Guosheng Song[1], Teng Liu[1], Xuan Yi[2], Kai Yang[2], Liang Cheng[1]*, and Zhuang Liu[1]*

[1]Institute of Functional Nano & Soft Materials (FUNSOM), Collaborative Innovation Center of Suzhou Nano Science and Technology, Soochow University, Suzhou, Jiangsu 215123, China

[2]School of Radiation Medicine and Protection & School for Radiological and Interdisciplinary Sciences (RAD-X), Jiangsu Provincial Key Laboratory of Radiation Medicine and Protection. Soochow University, Suzhou, Jiangsu, 21513, China

Email: zliu@suda.eud.cn; lcheng2@suda.edu.cn

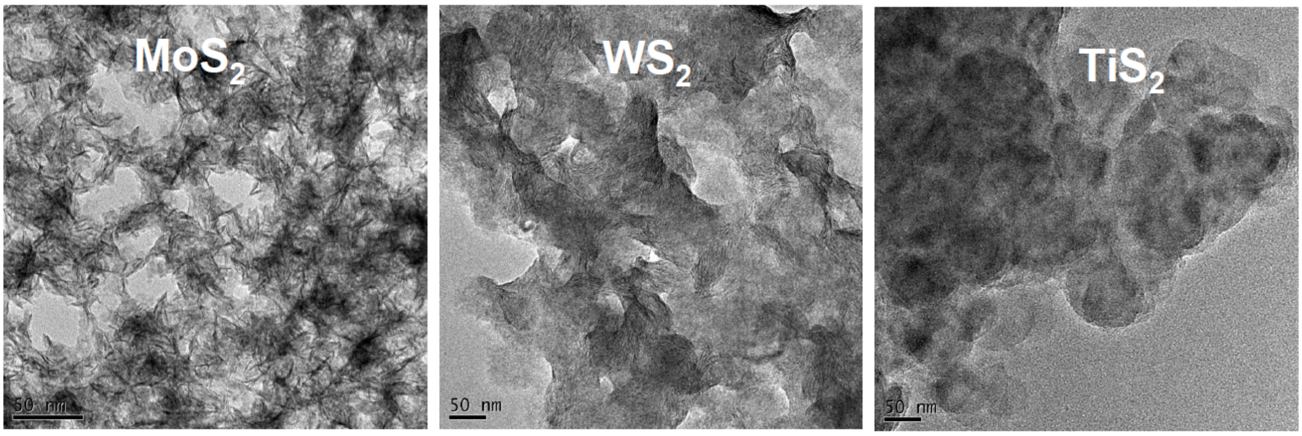

Supporting Figure S1 TEM images of the MoS_2, WS_2, and TiS_2 nanoflakes synthesized by the high-temperature thermal decomposition method.

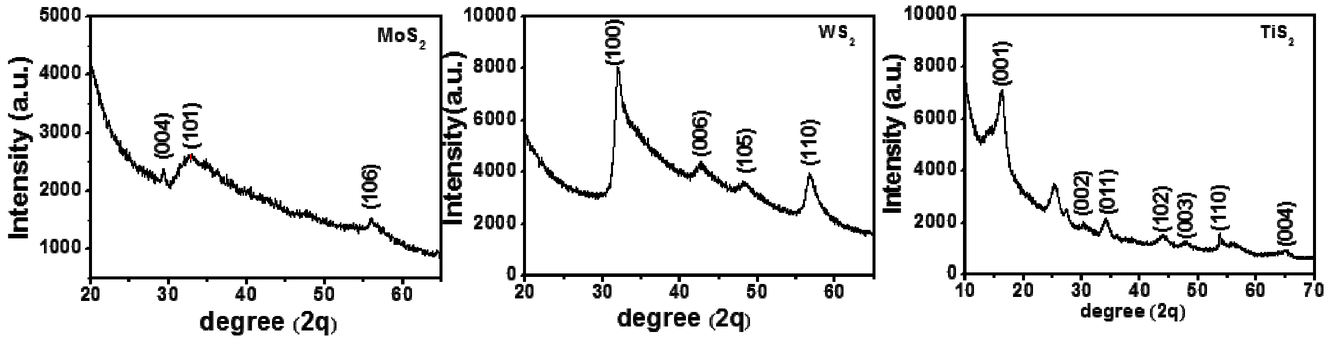

Supporting Figure S2. XRD spectra of the MoS_2, WS_2, and TiS_2 nanosheets under the ultrasonic decomposition each flakes.

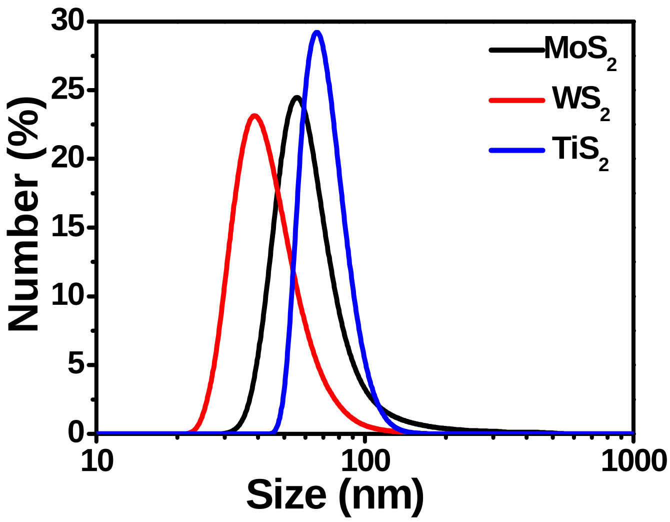

Supporting Figure S3 DLS size distribution of MoS_2, WS_2, and TiS_2 nanosheets in water.

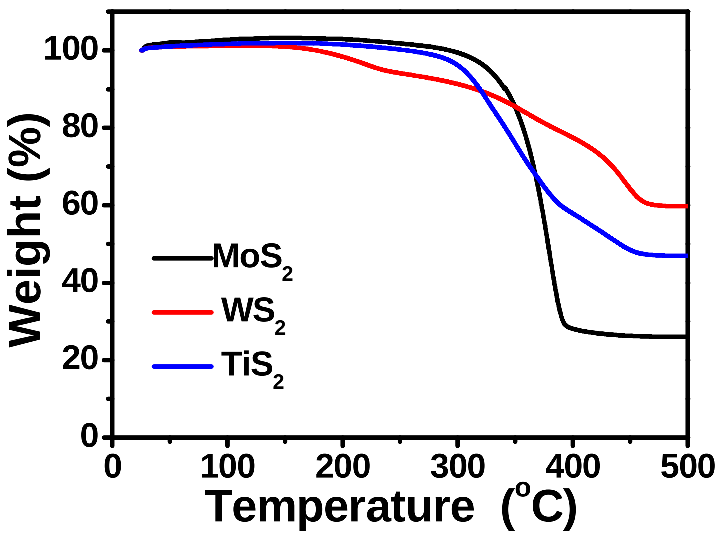

Supporting Figure S4 The weight percentage of MoS_2, WS_2, and TiS_2 in PEGylated MoS_2, WS_2, and TiS_2.

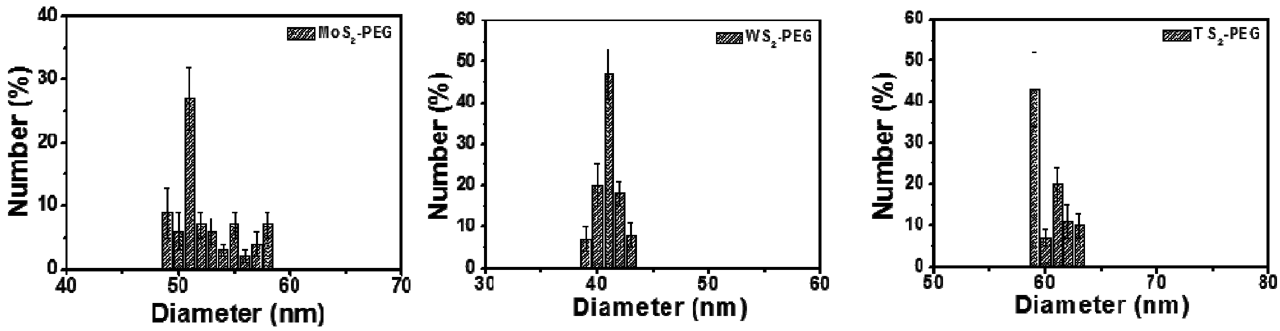

Supporting Figure S5 The size of PEGylated MoS_2, WS_2, and TiS_2 under TEM.

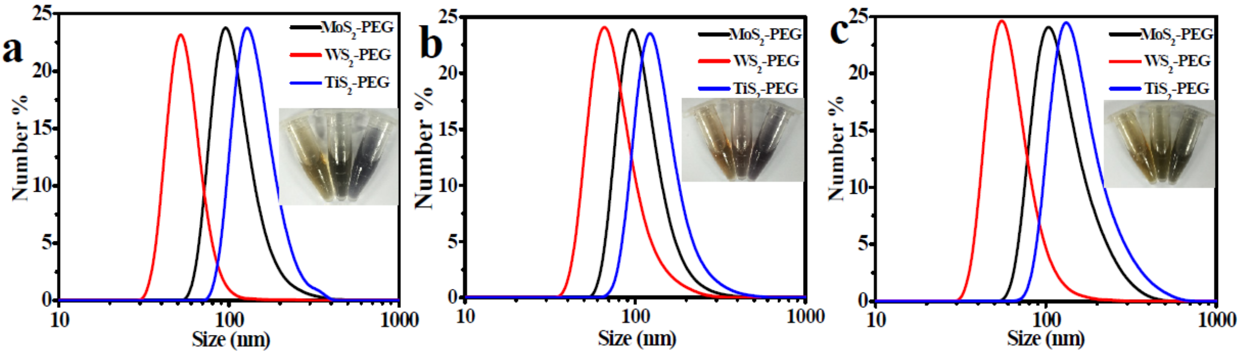

Supporting Figure S6 DLS size distribution of PEGylated MoS_2, WS_2, and TiS_2 in different solutions. **a**) Phosphate buffered saline (PBS). **b**) RMPI-1640 cell medium, and **c**) fetal bovine serum (FBS).

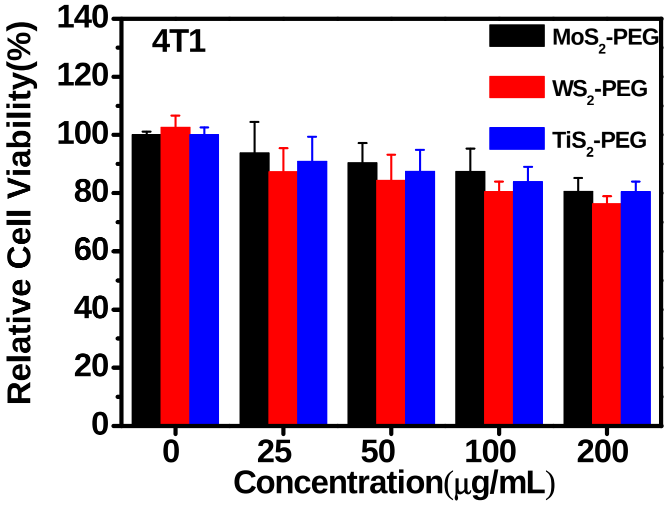

Supporting Figure S7 Relative cell viability of 4T1 cell line after incubation with PEGylated MoS2, WS2, and TiS2 respectively at different concentrations (in terms of TMDC weight concentrations) for 24 h.

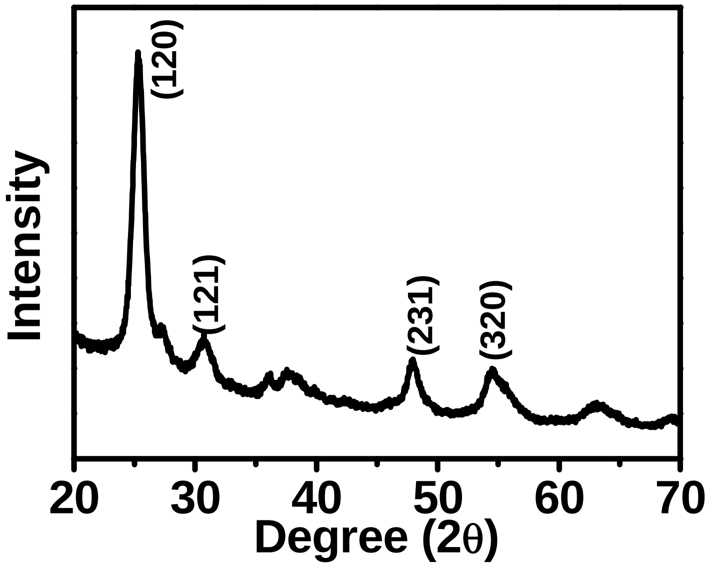

Supporting Figure S8 XRD spectrum of the PEGylated TiS_2 after 3 months standing in PBS solution at room temperature. It can be found the TiS_2 nanosheets were hydrolyzed to form TiO_2 nanostructures.
